# A comprehensive scoring system for the better prediction of bowel resection in pediatric intussusception

**DOI:** 10.1186/s12876-024-03243-6

**Published:** 2024-05-22

**Authors:** Bingshan Xia, Guoqiang Chen, Qianyang Liu, Chengwei Yan, Peng Lu, Chunbao Guo

**Affiliations:** 1 Department of Pediatrics, Women’s and Children’s Hospital, Chongqing Medical University, 120 Longshan Rd., Chongqing, 401147 P.R. China; 2 Department of Pediatrics, Yongchuan maternal and Child Health Hospital, Chongqing, P.R. China; 3 Department of Pediatric General Surgery, Chongqing University Three Gorges Hospital, Chongqing, P.R. China; 4 Department of Pediatrics, Chongqing health center for women and children, Chongqing, P.R. China; 5 Department of Pediatric General Surgery, Chongqing maternal and Child Health Hospital, Chongqing Medical University, Chongqing, P.R. China

**Keywords:** Intussusception, Scoring system, Bowel resection

## Abstract

**Background:**

Intussusception presents a significant emergency that often necessitates bowel resection, leading to severe complications and management challenges. This study aims to investigate and establish a scoring system to enhance the prediction of bowel resection necessity in pediatric intussusception patients.

**Methods:**

This retrospective study analyzed 660 hospitalized patients with intussusception who underwent surgical management at a pediatric hospital in Southwest China from April 2008 to December 2020. The necessity of bowel resection was assessed and categorized in this cohort. Variables associated with bowel resection were examined using univariate and multivariate logistic regression analyses. Based on these analyses, a scoring system was developed, grounded on the summation of the coefficients (β).

**Results:**

Among the 660 patients meeting the inclusion criteria, 218 required bowel resection during surgery. Bowel resection occurrence was linked to an extended duration of symptoms (Odds Ratio [OR] = 2.14; 95% Confidence Interval [CI], 1.03–5.23; *P* = 0.0015), the presence of gross bloody stool (OR = 8.98; 95% CI, 1.76–48.75, *P* < 0.001), elevated C-reactive protein levels (OR = 4.79; 95% CI, 1.12–28.31, *P* = 0.0072), lactate clearance rate (LCR) (OR = 17.25; 95% CI, 2.36–80.35; *P* < 0.001), and the intussusception location (OR = 12.65; 95% CI, 1.46–62.67, *P* < 0.001), as determined by multivariate logistic regression analysis. A scoring system (totaling 14.02 points) was developed from the cumulative β coefficients, with a threshold of 5.22 effectively differentiating infants requiring surgical intervention from others with necrotizing enterocolitis (NEC), exhibiting a sensitivity of 78.3% and a specificity of 71.9%.

**Conclusions:**

This study successfully identified multiple risk factors for bowel resection and effectively used a scoring system to identify patients for optimal clinical management.

**Supplementary Information:**

The online version contains supplementary material available at 10.1186/s12876-024-03243-6.

## Background

Intussusception, a prevalent gastrointestinal disorder in early childhood, predominantly impacts infants aged 4–10 months [1, 2]. The majority of occurrences in infants and toddlers are idiopathic, characterized by symptoms such as abdominal pain, vomiting, irritability, and currant jelly stools.

Nonsurgical reduction, encompassing hydrostatic or pneumatic techniques, is the preferred initial approach, demonstrating a success rate of 85–90% [3]. Surgical intervention is advised primarily when nonsurgical attempts fail or in the presence of peritonitis or perforation. The key focus during surgery is to assess bowel viability and maximize bowel length preservation. Delays in treatment can lead to extensive bowel loss and consequential severe complications, including long-term nutritional and developmental deficits. Despite efforts to prevent intestinal necrosis, complete avoidance of bowel resection and loss is not always possible. Various factors, including specific patient pathologies and surgeon expertise, have been identified as predictors of bowel resection [4, 5]. Additionally, systemic inflammatory markers like the lymphocyte-CRP ratio (LCR), platelet-lymphocyte ratio (PLR), and neutrophil-lymphocyte ratio (NLR) are proposed as valuable biomarkers for predicting inflammatory conditions such as mesenteric ischemia. Moreover, while multiple factors related to bowel resection have been studied, an integrated assessment combining radiographic, laboratory tests, and physical examination parameters is still lacking [6]. Prompt and precise identification of patients at risk for bowel resection is essential for timely and effective intervention.

In this study, we investigate the predictors of bowel resection in pediatric intussusception patients through a retrospective review of clinical data, which may contribute to improved outcomes.

## Methods

### Patient population

This collaborative, multidisciplinary program was dedicated to optimizing intussusception management. Participating institutions included the pediatric general surgery departments of Qingdao Maternity and Child Care Hospital, Yangchuan Maternal and Child Health Hospital, Chongqing Maternity and Child Care Hospital, and Chongqing Children’s Hospital. Medical data from patients diagnosed with intestinal intussusception across these hospitals were retrospectively reviewed from April 2008 to December 2020.

In our practice, patients initially undergo assessment via sonography, followed by further evaluation through air reduction enemas or surgical findings. Air reduction procedures, conducted promptly upon clinical or sonographic diagnosis, are performed by both surgeons and radiologists. Surgical management is pursued if air-enema reduction is unsuccessful. During exploratory laparotomy, assessing the viability of the affected bowel segment is crucial, following established operating room procedures. Ultimately, the decision to remove intestinal sections rests with the attending surgeon.

This study encompassed all intussusception cases that underwent surgical management, categorizing them into bowel resection and no-bowel resection groups. Inclusion criteria were an age range of over 1 month to under 3 years and being a first admission. Pathological lead points (PLPs) responsible for intussusception were excluded from the study. Due to its observational nature, patient consent was not required. Comprehensive reviews of available medical records were conducted, encompassing demographic, clinical features, and the entire spectrum of preoperative, intraoperative, and postoperative data. Additionally, the study explored the neutrophil-lymphocyte ratio (NLR) and lymphocyte-CRP ratio (LCR), to predict intestinal resection in intussusception patients.

### Statistical analyses

Data manipulation and statistical analysis were carried out using SPSS software (version 26, SPSS Corp, Chicago, IL). Initially, the Kolmogorov-Smirnov test was applied to evaluate data distribution. Continuous variables with normal distribution were presented as means ± SD and analyzed using the independent Student’s t-test. In contrast, abnormally distributed continuous variables were displayed as medians (range) and assessed with the Mann-Whitney U-test. Categorical variables were reported as frequencies (percentages) and examined using either the chi-square test or Fisher’s exact test, accompanied by relative risk estimation.

To evaluate the diagnostic utility of each predictor for bowel resection, receiver operating characteristic (ROC) curve analysis was employed, and the area under the ROC curve (AUC) was assessed to establish the optimal predictive value of specific measurements. Binary multivariate unconditional logistic regression analysis was utilized, incorporating comprehensive clinical, ultrasonographic, radiographic, and laboratory variables (P-value below 0.05 in univariate analysis). A prediction scoring system was developed based on the total coefficient (β) of each variable, with the variable’s contribution derived from its coefficient (β) relative to the total scoring system. Optimal cut-off values for the total scores were ascertained using Youden’s index method to maximize the performance of each parameter and the overall scoring system. A P-value of less than 0.05 was considered statistically significant.

## Results

Throughout the study period, April 2008 to December 2020, my institute conducted air-enema reduction procedures on 8,138 intussusception cases. Of these, 739 patients underwent surgical laparotomy following unsuccessful air-enema reduction, resulting in an air-enema reduction success rate of 90.9%.

**Fig. 1:**
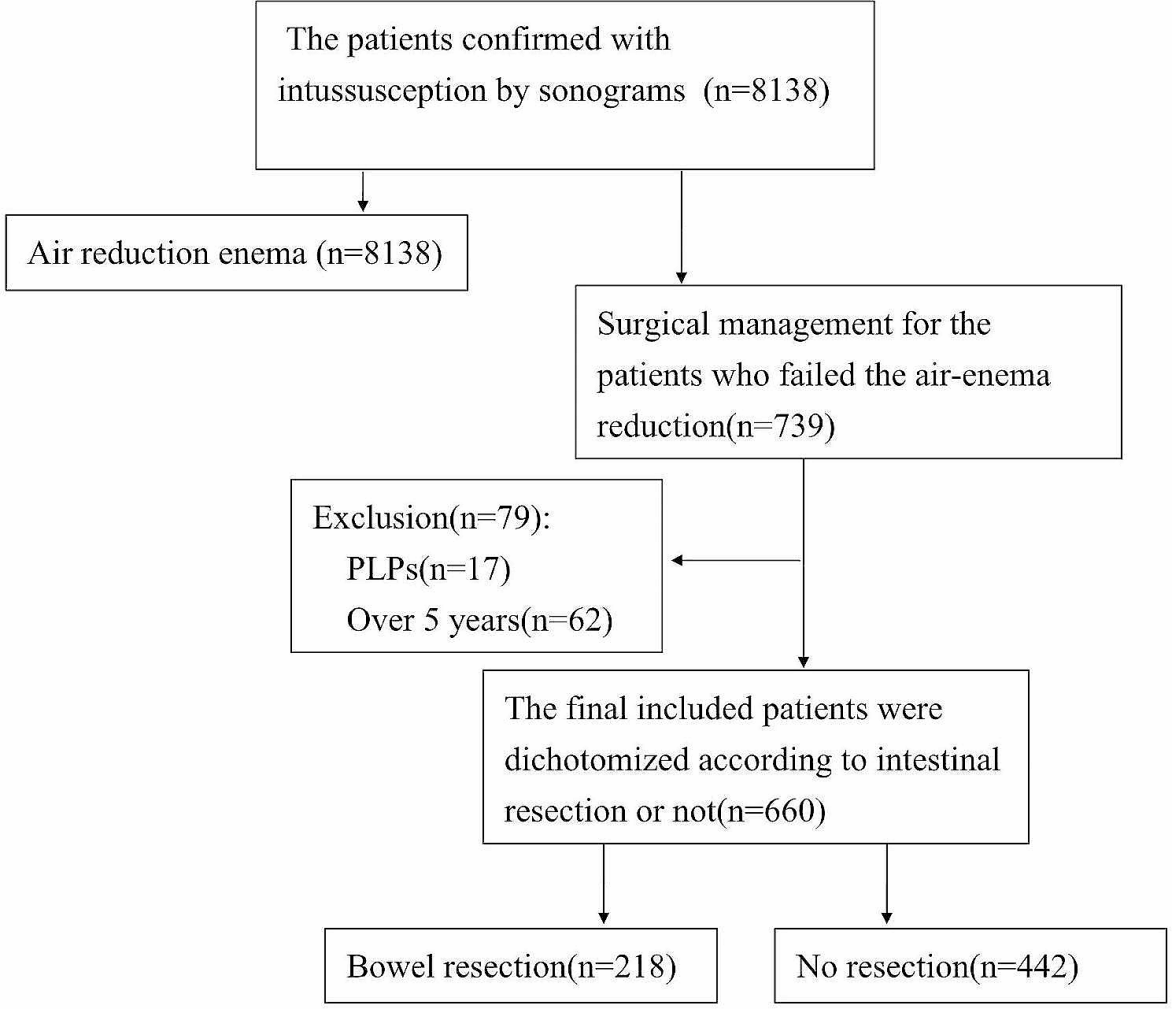
Study flow chart for selection of the study population

This study involved the collection and analysis of detailed medical data from patients who received surgical management for intussusception. Of these cases, 62 patients older than 5 years were excluded from the study. Additionally, 17 cases identified with pathological lead points (PLPs) during surgery were also excluded. Ultimately, 660 patients satisfied the inclusion criteria and were enrolled in the study. These patients were categorized into two groups: 218 in the intestinal resection group, who underwent bowel resection, and 442 in the non-intestinal resection group, who had laparotomies without bowel resection. A flow chart detailing the inclusion and exclusion criteria, and the composition of the final cohort, is illustrated in Fig. 1.

Table 1 provides a detailed summary and presentation of the clinical characteristic variables between the two groups. Univariate analyses revealed a significant increase in gross bloody stool (*P* < 0.001) and vomiting (*P* = 0.008) in the bowel loss group, indicating a more severe form of intussusception compared to the group without bowel loss. Regarding patient age, the bowel loss group was notably younger (*P* = 0.013). Additionally, this group experienced a longer duration of symptoms upon admission (*P* = 0.0063). Inflammatory parameters, including WBC, CRP, PLR, and LCR, were markedly higher in patients with bowel loss. Ultrasound assessment was used to evaluate the location of intussusception, revealing a greater likelihood of bowel resection as the intussusception extended farther. However, no significant differences were observed in BMI, recurrence, and reduction pressure between the groups.

**Table 1:** Characteristics of children with intussusception under bowel resection or not

Characteristics	No resection *n* = 442	Bowel resection *n* = 218	*P* value
Male, n (%)	302	143	
Female, n (%)	140	65	0.49
Age, median (IQR)	21.88(3.2–61.6)	15.73(4.1–60.9)	0.013
Duration of symptoms(h), median (IQR)	16 (2–68)	32 (5–92)	0.0063
BMI (Kg/m^2^, mean ± SD)	18.9 ± 2.9	19.6 ± 3.6	0.17
Symptoms, n (%)			
Intermittent crying	419(94.8%)	212(97.2%)	0.104
Vomiting	378(85.5%)	201(92.2%)	0.008
Bloody stool	323(73.1%)	187(85.8%)	<0.001
Fever > 37.5 °C (%)	156(35.3%)	86(39.4)	0.17
Recurrence	35(7.9%)	8(3.7%)	0.024
WBC count(>12.010^9^ /L), n (%)	275(62.2)	159(72.9)	0.004
C-reactive protein(>8.0 mg/L), n (%)	317(71.7)	183(83.9)	<0.001
PLR(> 188.5), n (%)	288(65.2)	175(80.3)	0.027
LCR(< 0.121), n (%)	306(69.2)	198(90.8)	< 0.001
Abdominal mass, n (%)	219(49.5)	121(55.5)	0.087
Pressure (Kp, mean ± SD)	8.94 ± 1.42	11.04 ± 1.59	0.36
Location of intussusception, n (%)			
Ascending colon	376(85.1)	107(49.1)	<0.001
Transverse colon	57(12.9)	84(38.5)	<0.001
Descending colon	9(2.0)	11(5.0)	0.033
Sigmoid colon	0(0)	16(7.3)	<0.001

LCR = lymphocyte–CRP ratio; PLR = neutrophil–lymphocyte ratio; Recurrence: Intussusception occurred before admission

Receiver Operating Characteristic (ROC) analysis was conducted for all mentioned parameters and the results are detailed in Table 2. Area Under the Curve (AUC) evaluations revealed statistically significant predictors of bowel resection, which included a prolonged duration of illness, presence of bloody stool, abdominal muscle rigidity, elevated white blood cell (WBC) count, and the extent of intussusception. These factors were found to predict bowel resection with relatively high accuracy.

**Table 2:** ROC curve results and sensitivity, specifcity values

	Age	Duration of illness	Fever > 37.5 °C	Bloody stool	LCR	CRP	WBC	Extent of intussusceptions
p values	0.074	< 0.001	0.053	≤ 0.001	0.0064	0.037	0.0018	< 0.001
Cut-off	8.72	28.64	/	/	< 0.121	8.0	12.0	/
Sensitivity (95% CI)	0.593	0.698	0.428	0.769	0.431	0.464	0.612	0.797
Specificity (95% CI)	0.412	0.846	0.510	0.891	0.597	0.524	0.478	0.854
Accuracy	50.8	76.9	54.3	81.5	63.8	50.2	54.8	82.7
NPV	0.84	0.86	0.78	0.94	0.87	0.90	0.92	0.89
PPV	0.46	0.49	0.51	0.63	0.64	0.63	0.71	0.62

Multivariable logistic regression was utilized to identify independent risk factors for intestinal necrosis in intussusception patients, based on potential variables identified in the univariate analysis. The multivariate analysis identified several independent risk factors: duration of symptoms (Odds Ratio [OR] = 2.14; 95% Confidence Interval [CI], 1.03–5.23; *P* = 0.0015), presence of gross bloody stool (OR = 8.98; 95% CI, 1.76–48.75, *P* < 0.001), elevated C-reactive protein levels (OR = 4.79; 95% CI, 1.12–28.31, *P* = 0.0072), lactate clearance rate (LCR) (OR = 17.25; 95% CI, 2.36–80.35; *P* < 0.001), and the location of intussusception (OR = 12.65; 95% CI, 1.46–62.67, *P* < 0.001) (Table 3). Subsequently, a 14.02-point scoring system was developed based on the β sums from each factor. Factors that received a low score were omitted. For each variable, a score was assigned when the parameter was present. A cut-off value of 5.22 was established to differentiate patients requiring bowel resection, demonstrating a sensitivity of 78.3% and a specificity of 71.9%.

**Table 3:** Multivariate models for the bowel resection

	Regression coefficient(β)	OR	95%CI	*P* value
Position of intussusceptions	2.74	12.65	1.46 ~ 62.67	<0.001
Bloody stool	3.46	8.98	1.76 ~ 48.75	<0.001
CRP	2.44	4.79	1.12 ~ 28.31	0.0072
LCR	3.75	17.25	2.36 ~ 80.35	<0.001
Duration of illness	1.63	2.14	1.03 ~ 5.23	0.0015

Total score = 14.02; Cut off = 5.22; >5.22 means bowel loss; <5.22 means non bowel loss

## Discussion

In this current study, we explored various variables associated with bowel loss, including clinical symptoms, physical examinations, laboratory tests, and ultrasonographic findings. Bloody stool, prolonged illness duration, fever, abdominal muscle rigidity, severe inflammation, and the location of intussusception emerged as independent predictors for bowel loss in intussusception patients. Additionally, we developed a new scoring system for bowel resection in these patients, integrating as many variables as possible. To the best of our knowledge, this study represents the first report of a precise diagnostic benchmark that definitively predicts the need for bowel resection in pediatric intussusception cases.

Intussusception is a common cause of bowel resection in infants and young children [7, 8]. It is well-established that prompt identification and management are crucial for reducing the incidence of intestinal necrosis resulting from intussusception. However, a significant number of patients experience delays in diagnosis due to the rapid progression of intussusception in cases lacking classic early-stage characteristics [9–11]. In this study, among the 660 patients who underwent surgery, 33.0% of those with intussusception required intestinal resection, constituting 2.7% (218/8,138) of all intussusception cases managed. Other centers in China reported a bowel resection rate of 24.1% (76/316) in patients with intussusception who underwent exploratory laparotomy [6]. In Canada, 28% of cases involving operations required resection [12]. Differences in basic characteristics among these reports may explain the higher proportion of intestinal loss in the latter study. For instance, I preferred and made efforts to perform air reduction, attempting it twice in some cases. Furthermore, management decisions were made by attending surgeons in various institutes with varying criteria, contributing to these disparities [13]. More than 30% of patients in this series exhibited intussusceptum necrosis, primarily due to mesenteric entrapment, indicating that a substantial number of cases in this study were managed relatively late. It is plausible to infer that earlier intervention could have benefited these patients.

In this study, we conducted a retrospective analysis of clinical data in patients with intussusception, aiming to investigate a comprehensive set of readily available clinical characteristics associated with bowel loss. Many of the variables examined in this study have been previously assessed, and no single predictor demonstrated clear superiority [7, 14, 15]. Our findings revealed that the most predictive factors for bowel resection included the presence of bloody stool, prolonged disease duration, and the location of intussusceptions. Furthermore, we observed a correlation between bowel loss and symptoms such as abdominal distension, abdominal muscle rigidity, as well as elevated levels of white blood cells (WBC) and C-reactive protein (CRP), indicative of severe inflammation. Given the high incidence of bowel loss associated with these variables, timely surgical consultation should be considered, and early surgical intervention may prove beneficial in preventing bowel necrosis, even in cases where air reduction is not attempted [16, 17].

In this study, a longer duration of symptoms emerged as a predictive factor for bowel loss in pediatric intussusception cases. This finding aligns with results from previous studies on the management of childhood intussusceptions, where the duration of the disease was associated with surgical intervention and bowel loss. This suggests that intussusception cases with prolonged symptom duration should be carefully considered for surgical intervention, particularly in the era of laparoscopy [5, 18]. Bloody stool typically represents a later sign in the progression of the disease, often leading patients to seek medical attention after some delay, posing a challenge in intussusception management. Early, nonspecific symptoms such as refusal to eat and episodes of crying may go unnoticed by parents, potentially contributing to delayed medical care. Consequently, the reported duration of illness may underestimate the true duration in many cases [19].

Radiation-free ultrasound is the preferred and initial diagnostic choice for evaluating intussusception, enabling the precise localization of the intussusception site [20]. Our findings indicate that the deeper the intussusception, the higher the risk of bowel loss during intussusception management. It’s important to note that air reduction itself can pose a risk factor for intestinal necrosis, although this study cannot directly demonstrate the impact of air reduction on intestinal loss. This is because as gas enters from the hepatic hilum and reaches 12 kPa, it further compresses the intestinal canal and exacerbates mesenteric ischemic injury. In the era of laparoscopy, surgical intervention should be seriously considered when dealing with deep intussusception to prevent worsening ischemic injury and the need for intestinal resection.

Due to the limitations of clinical examinations and the relatively low accuracy of ultrasonography in diagnosing bowel ischemia or necrosis, relying solely on clinical exams, laboratory assessments, or ultrasonographic evaluations is not a comprehensive approach to predict patient outcomes. In our the present study, we have developed an integrated scoring system that incorporates clinical, laboratory, and ultrasonographic variables to predict the likelihood of bowel resection [21]. Notably, our study stands out for its utilization of complex mathematical processing, revealing significant variations in the weights of these variables. We propose a cut-off value of 5.22 as an indicative threshold for potential surgical intervention. To the best of our knowledge, this study holds significance as it provides pediatric surgeons with a rapid and precise prediction tool for suspected cases requiring bowel resection.

In various clinical settings, the Lymphocyte-CRP Ratio (LCR) has been identified as a rapid indicator reflecting systemic inflammatory responses [22]. Our ROC analysis results revealed that, among all the parameters examined, LCR stood out as a robust inflammatory marker significantly associated with intestinal resection. The intestinal ischemia is associated with inflammatory responses, which should be indicated by LCR value, a combination of immunological predictor [23, 24]. Peripheral lymphocytes play a vital role in the host’s cytotoxic immune response to intestinal microflora, while C-reactive protein (CRP) alone serves as an effective inflammatory marker [25]. As demonstrated in this study, a low LCR value indicates an intensified systemic inflammatory response or impaired immunological response in patients with intussusception, serving as a valuable indicator for assessing intestinal ischemia. While LCR has been investigated in numerous inflammatory conditions, our study is the first, to our knowledge, to report its use in predicting the need for intestinal resection in cases of intussusception.

The present research exhibits several potential limitations that necessitate consideration when attempting to generalize the current findings. Firstly, it is essential to acknowledge the retrospective nature of this study, which inherently carries a risk of selection bias due to the data collection methodology. Furthermore, the study’s extended duration may have introduced management bias, potentially leading to confounding factors. Additionally, variations in protocols regarding surgical intervention decision-making, which were based on individual surgeon experience rather than randomization, may have influenced the results. Consequently, it is crucial not to haphazardly interpret the current data as a definitive justification for aggressive surgical interventions. To establish a more robust foundation, future multicenter prospective clinical trials with standardized criteria for bowel resection should be conducted to verify the optimal timing of interventions.

## Conclusions

In summary, the present study detected several clinical variables that could clearly separate patients with potential bowel loss, which should be considered for early referral to paediatric surgical centres to prevent gastrointestinal loss.

### Electronic supplementary material

Below is the link to the electronic supplementary material.

Supplementary Material 1

## Data Availability

The datasets used and/or analysed during the current study are available from the corresponding author on reasonable request.

## Abbreviations

AUC: the area under the ROC curve
CRP: C-reactive protein
LCR: lymphocyte-CRP ratio
NEC: necrotizing enterocolitis
NLR: neutrophil-lymphocyte ratio
PLR: platelet-lymphocyte ratio
PLPs: pathological lead points
ROC: receiver operating characteristic
WBC: white blood cells
