# Supplementary material for: A comprehensive scoring system for the better prediction of bowel resection in pediatric intussusception
Source: BMC Gastroenterol. 2024 May 22;24:180. doi: 10.1186/s12876-024-03243-6 (PMC11110355; doi:10.1186/s12876-024-03243-6)

## Slide 1
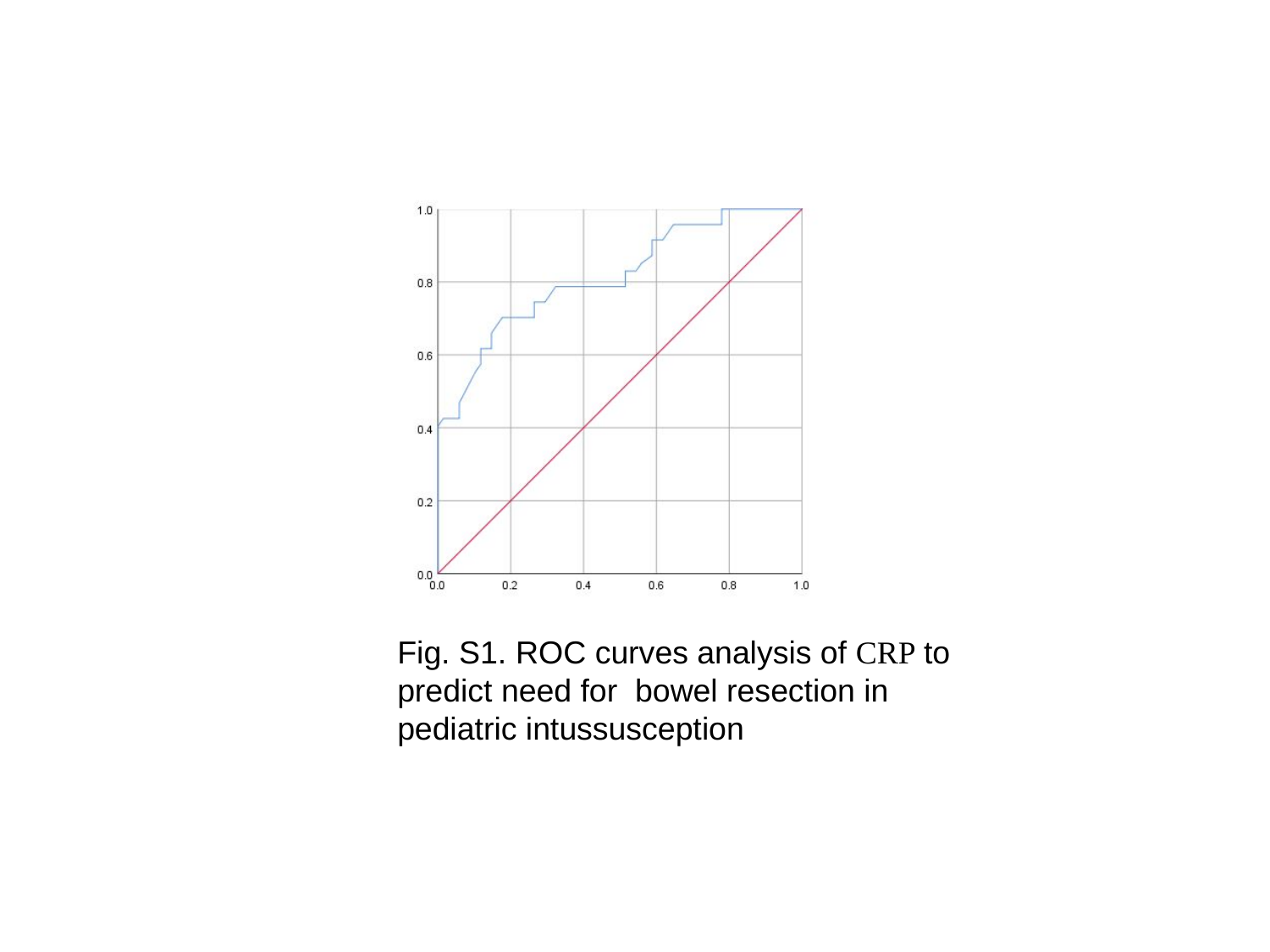

Fig. S1. ROC curves analysis of CRP to predict need for bowel resection in pediatric intussusception

## Slide 2
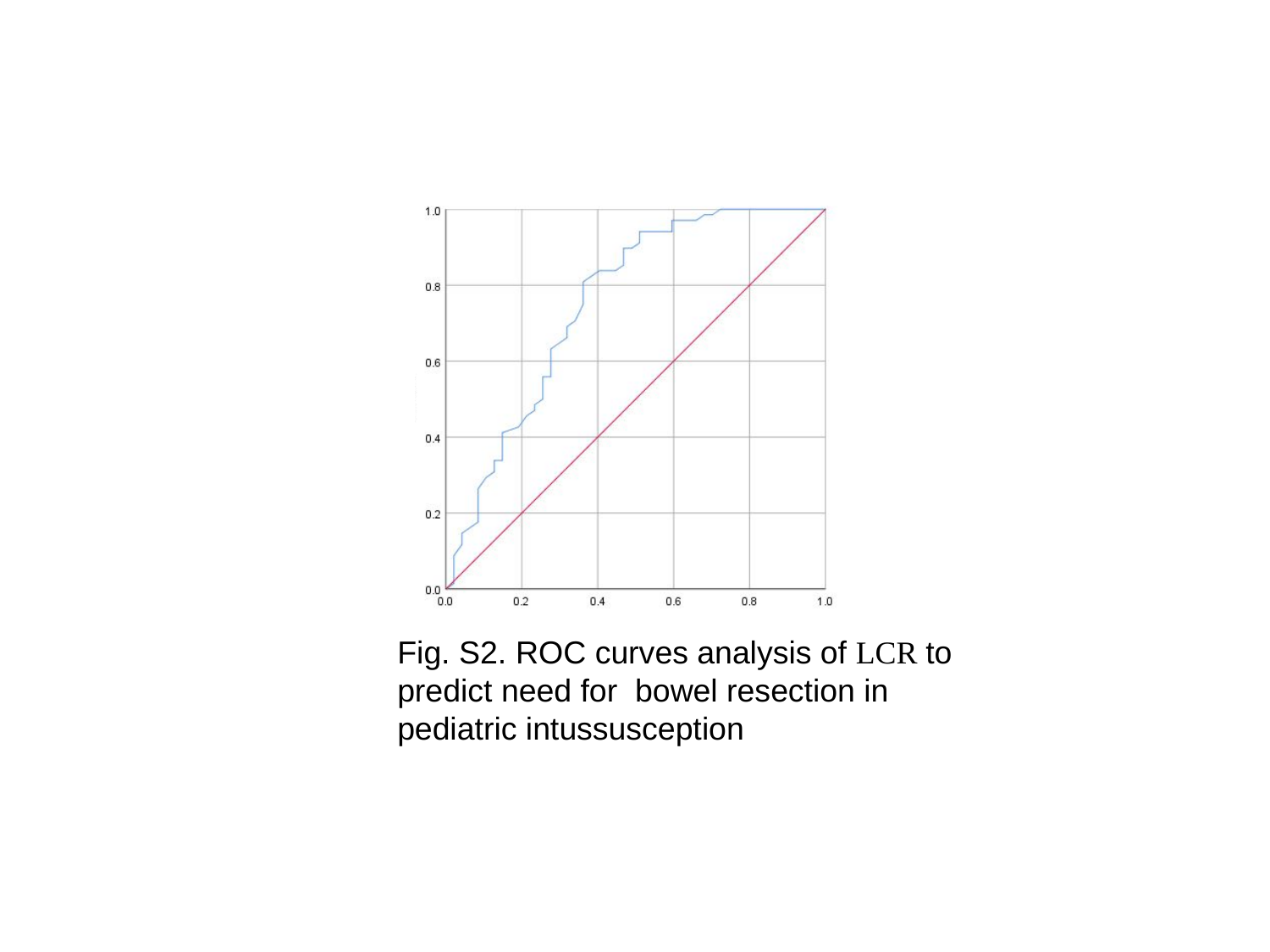

Fig. S2. ROC curves analysis of LCR to predict need for bowel resection in pediatric intussusception

## Slide 3
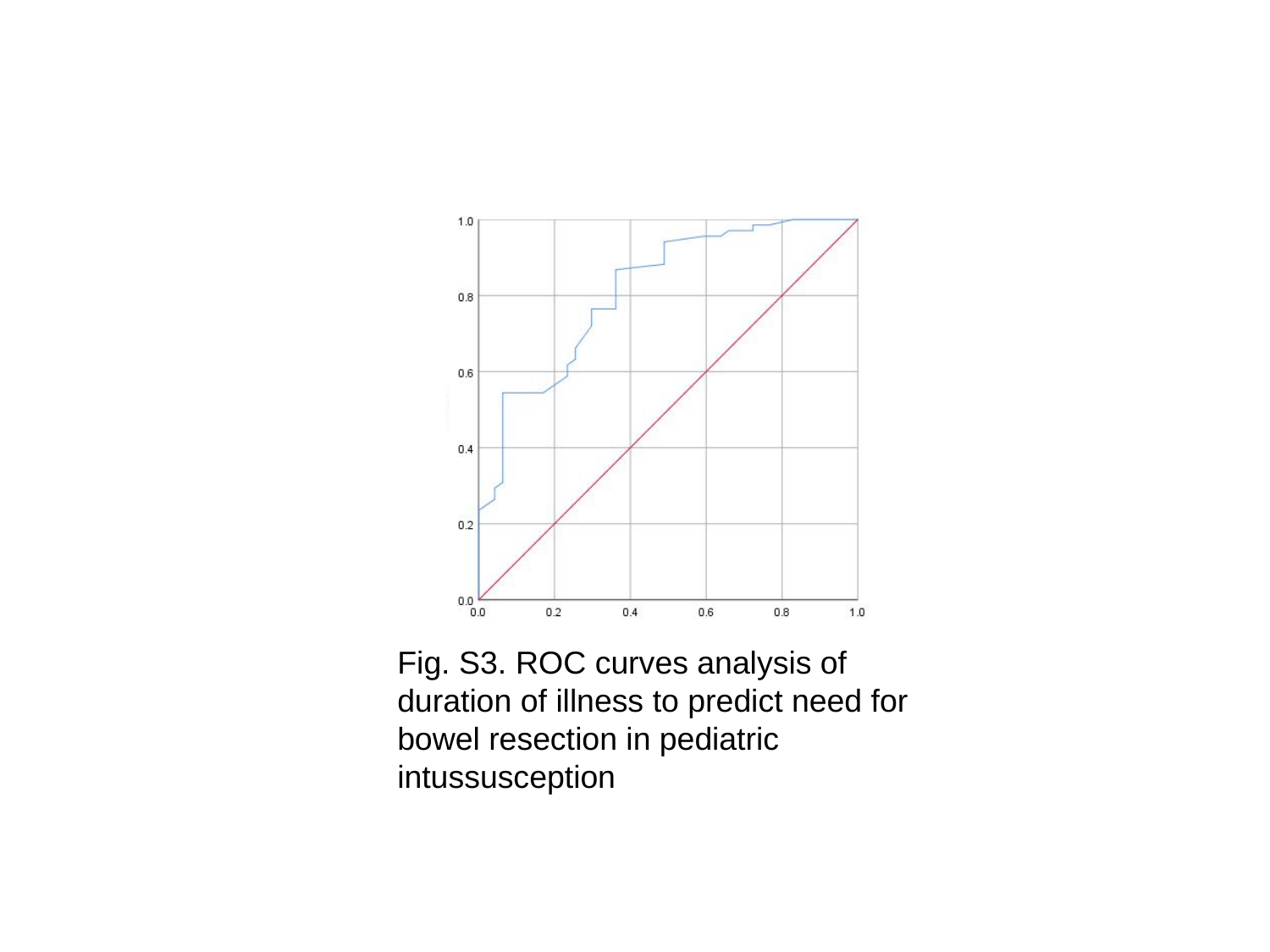

Fig. S3. ROC curves analysis of duration of illness to predict need for bowel resection in pediatric intussusception

## Slide 4
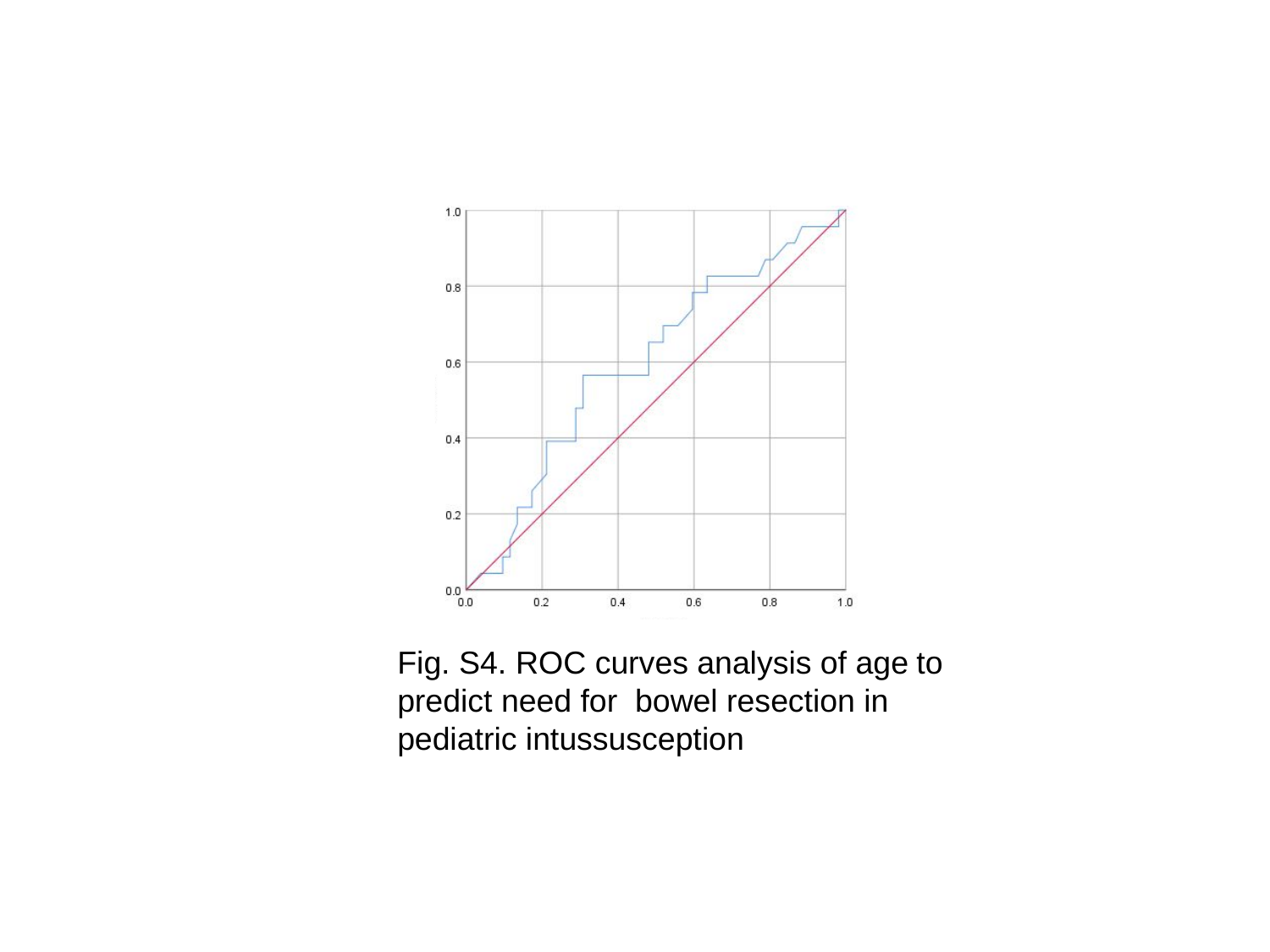

Fig. S4. ROC curves analysis of age to predict need for bowel resection in pediatric intussusception

## Slide 5
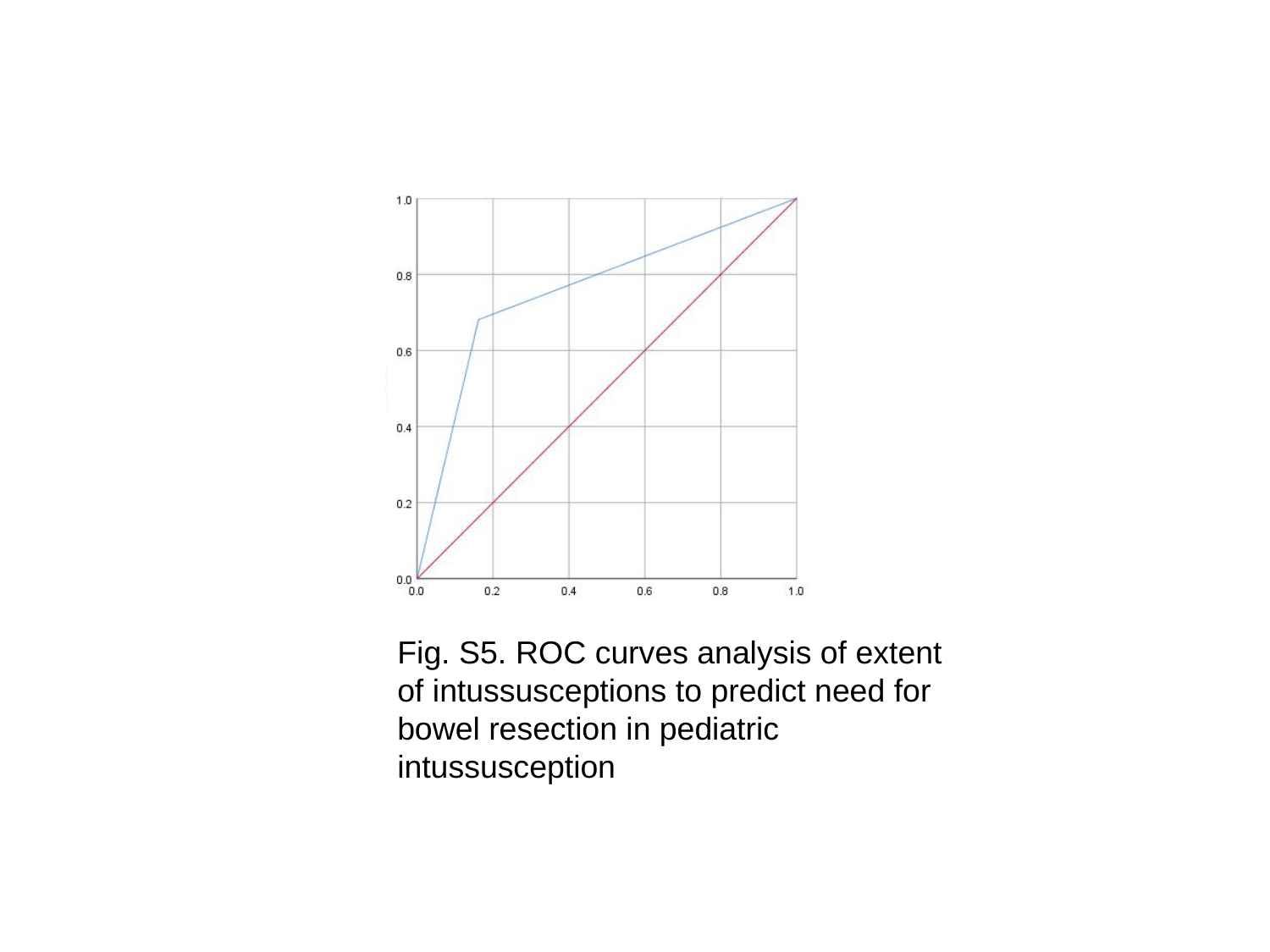

Fig. S5. ROC curves analysis of extent of intussusceptions to predict need for bowel resection in pediatric intussusception

## Slide 6
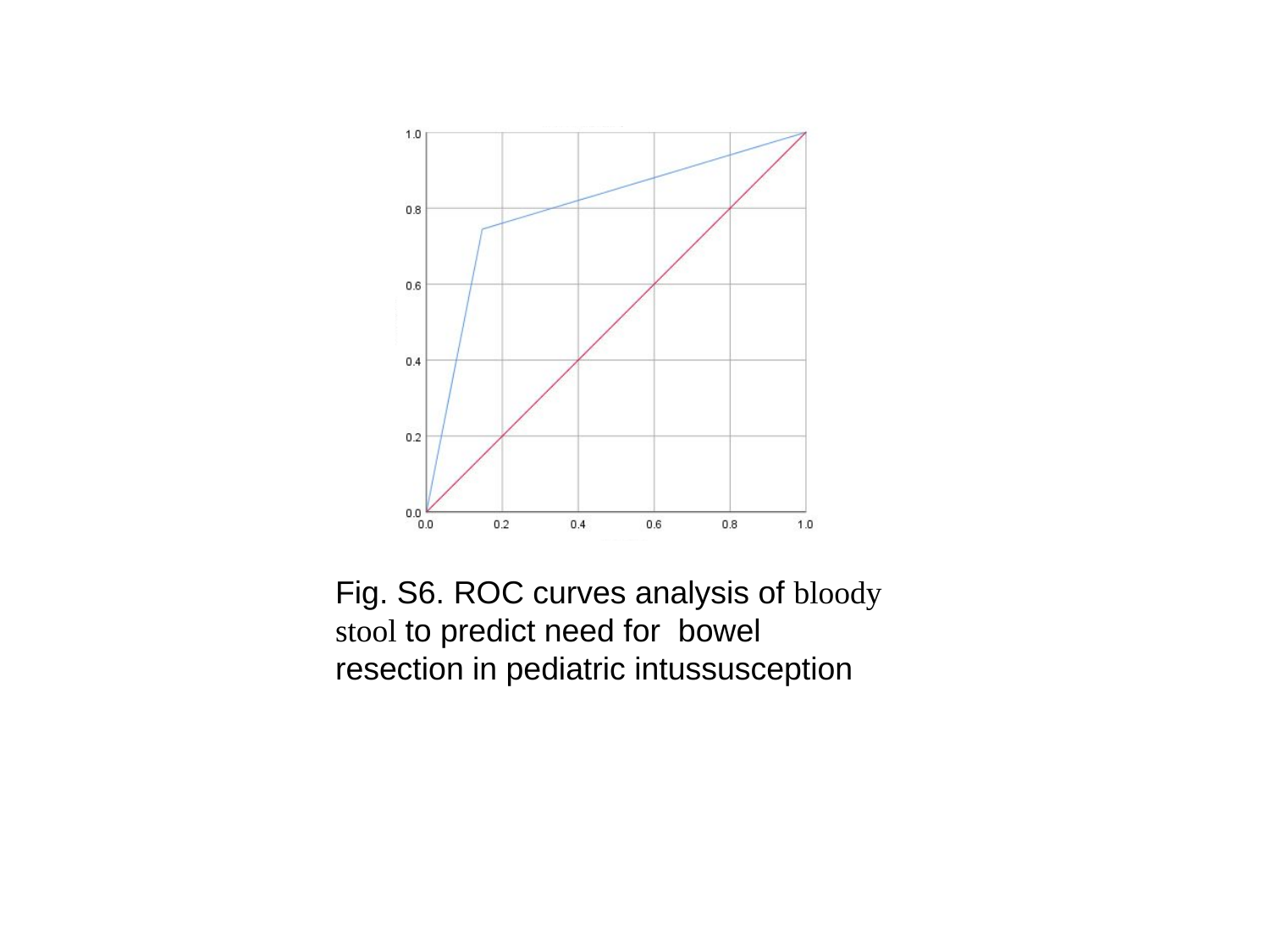

Fig. S6. ROC curves analysis of bloody stool to predict need for bowel resection in pediatric intussusception

## Slide 7
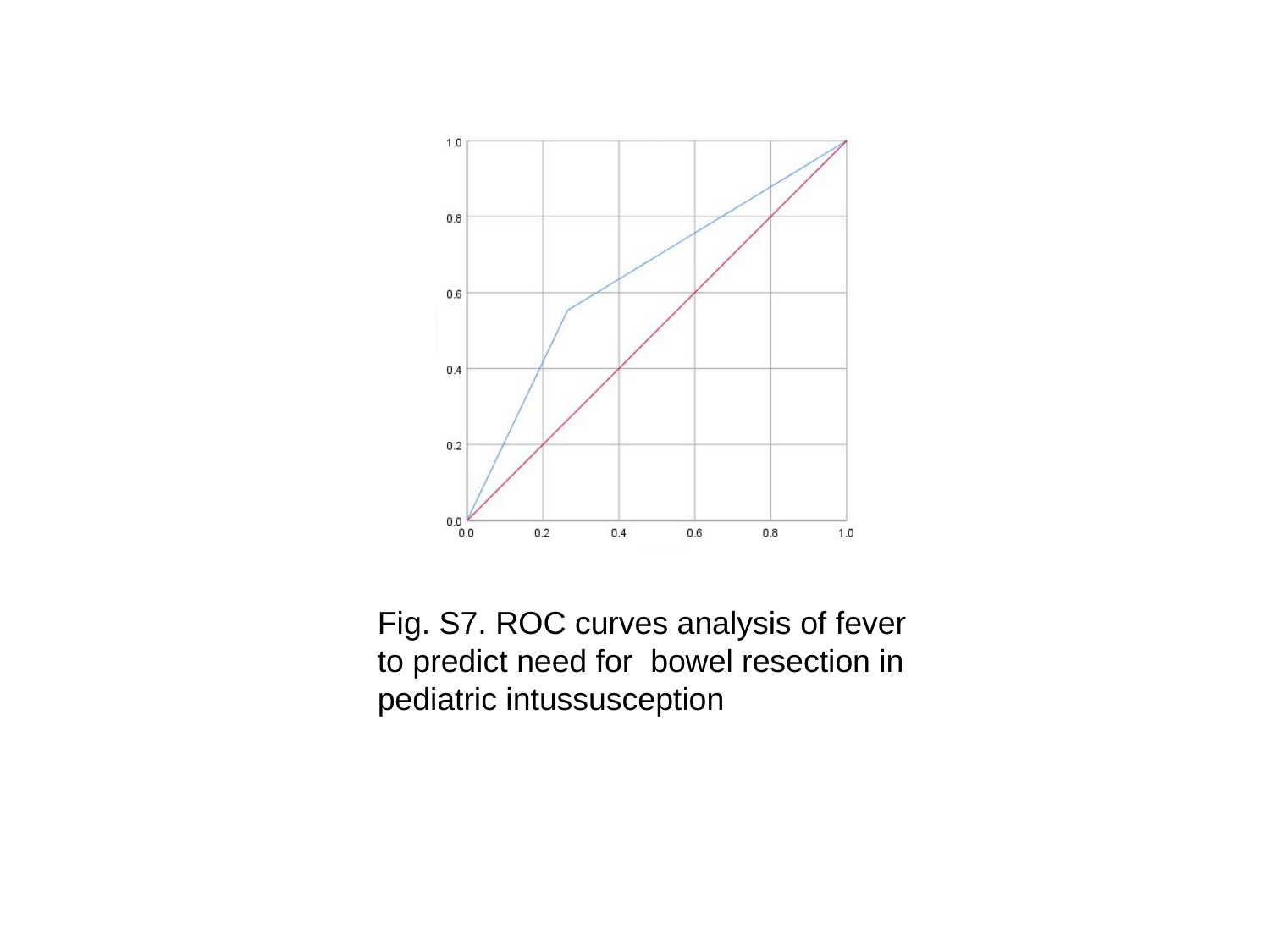

Fig. S7. ROC curves analysis of fever to predict need for bowel resection in pediatric intussusception

## Slide 8
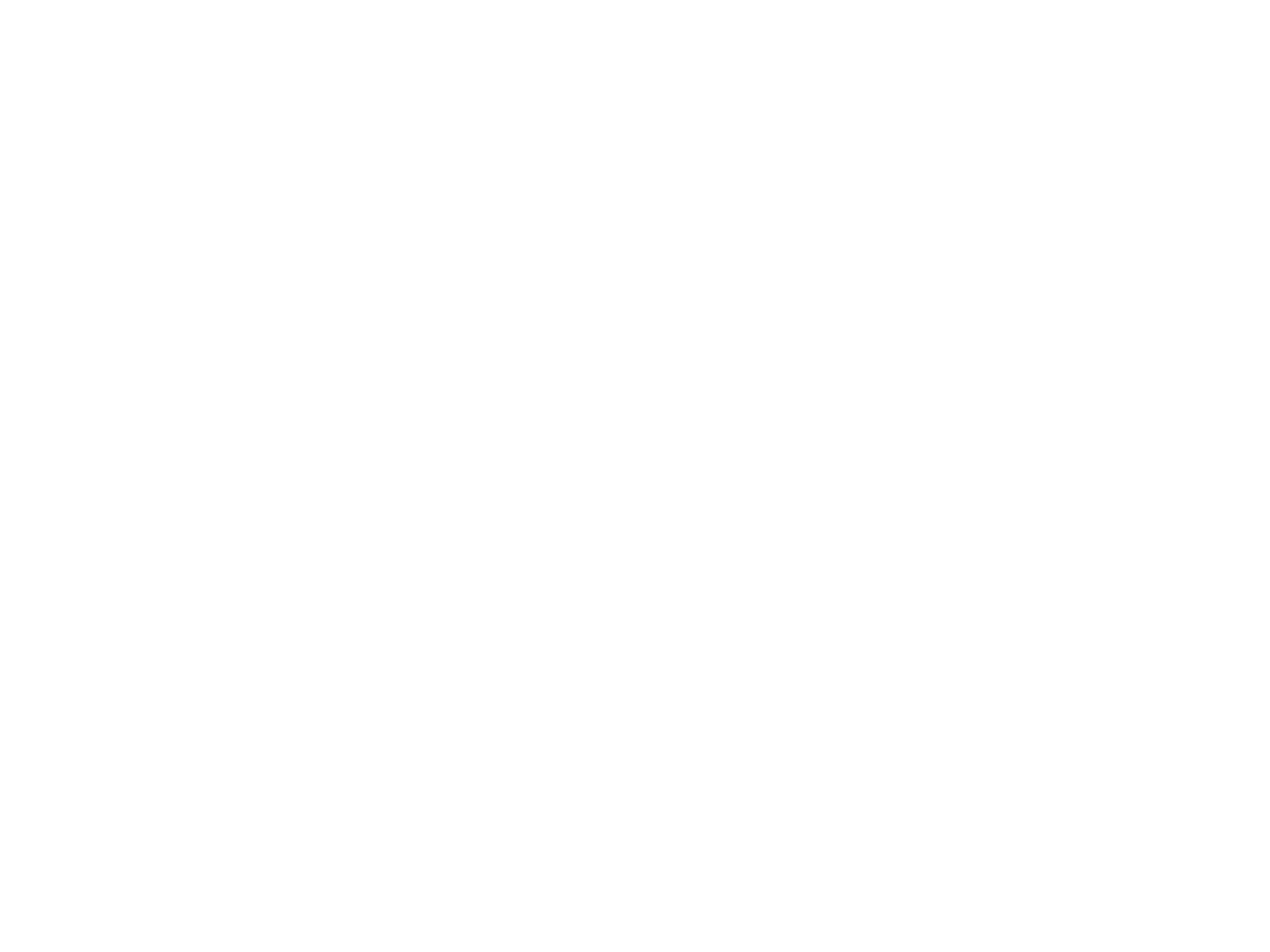

Supplement: Supplementary file 1 — Supplementary Material 1 [file 12876_2024_3243_MOESM1_ESM.pptx]
